# Compliance with National and International Guidelines in the Treatment of Nonmotor Symptoms in Late-Stage Parkinson's Disease

**DOI:** 10.1155/2023/6667339

**Published:** 2023-10-10

**Authors:** Kristina Rosqvist, Per Odin

**Affiliations:** ^1^Lund University, Faculty of Medicine, Department of Clinical Sciences Lund, Division of Neurology, Lund, Sweden; ^2^Skåne University Hospital, Department of Neurology, Rehabilitation Medicine, Memory and Geriatrics, Lund, Sweden; ^3^Department of Neurology, Klinikum Bremen-Nord, Bremen, Germany

## Abstract

**Background:**

National as well as international Parkinson's disease (PD) treatment guidelines are available to guide clinicians. Previous research has shown that nonmotor symptoms (NMS) are pronounced in late-stage PD and has suggested that current treatment is insufficient and could be improved.

**Objectives:**

The aim of this study was to investigate to which degree the national and international treatment guidelines are followed in the treatment of NMS in late-stage PD.

**Methods:**

This Swedish cohort was part of the Care of Late-Stage Parkinsonism (CLaSP) study. Late-stage PD was defined as Hoehn and Yahr stages IV-V in “on” and/or ≤50% on the Schwab and England Activities of Daily Living (ADL) scale. NMS were assessed with the NMS scale (NMSS), cognition with the Mini-Mental State Examination (MMSE), and depressive symptoms with the Geriatric Depression Scale (GDS-30). Symptomatic individuals were defined as ≥ 6 on an item of the NMSS; for dementia, a cutoff of ≤18 on the MMSE; for depression, a cutoff of ≥10 on the GDS.

**Results:**

All 107 participants exhibited NMS to various degrees and severities; the median NMSS score was 91. Among symptomatic individuals, for depressive symptoms, 37/63 (59%) were treated with antidepressants; for hallucinations and delusions, 9/18 (50%) and 5/13 (38%) were treated with antipsychotics; and for dementia, 9/27 (33%) were treated with rivastigmine and 1 (4%) was treated with donepezil. For orthostatic hypotension, 11/19 (58%) with lightheadedness and 7/8 (88%) with fainting were treated with antihypotensives; for sialorrhea, 2/42 (5%) were treated with botulinum toxin; and for constipation, 19/35 (54%) were treated with laxatives. For insomnia, 4/16 (25%) were treated with hypnotics, and for daytime sleepiness, 1/29 (3%) was treated with psychostimulants.

**Conclusions:**

The present analyses suggest a need for clinicians to further screen for and treat NMS. Optimizing treatment of NMS according to the national and international treatment guidelines may improve symptomatology and enhance quality of life in late-stage PD.

## 1. Introduction

National as well as international guidelines for the treatment of Parkinson's disease (PD) are available to guide clinicians to improve and optimize equal treatment and care for patients with PD [1–3].

In late-stage PD, i.e., Hoehn and Yahr (HY) stages IV-V [4], motor and nonmotor symptoms (NMS) are pronounced [5–8] and quality of life (QoL) is often reduced [9]. Previous research has suggested that current treatment in late-stage PD remains insufficient [6, 10] and could be improved.

Several studies have indicated that NMS may have a higher impact on QoL than motor symptoms [11–16]. It is therefore of high importance that NMS are screened for and adequately treated, not least in the late stage of the disease.

NMS include neuropsychiatric symptoms such as depressive symptoms, anxiety, apathy, and psychosis [17]; autonomic dysfunction such as orthostatic hypotension (OH), constipation, and difficulty swallowing; sleeping disorders, pain and fatigue [16, 18]. We have previously shown that a large range of NMS and particularly neuropsychiatric symptoms are common and pronounced in late-stage PD [8, 19]. Assessed by the NMS scale (NMSS) [20], the highest scores are found within the domain mood/apathy [8]. Multinational analyses have further confirmed that the most clinically relevant symptoms are apathy, depression, and anxiety [19]. Fatigue is another common and pronounced symptom among late-stage PD patients [6]. Multivariable linear regression analyses of multicenter data from a large cohort of late-stage patients across Europe showed that lower NMSS scores in the domains of mood/apathy, attention/memory, gastrointestinal, and sleep/fatigue were associated with better health-related QoL [9]. NMS affect a large proportion of people in late-stage PD [6, 15], with various symptoms occurring in >80% of the individuals [8]. The frequency of NMS increases with disease severity [11].

The International Parkinson and Movement Disorder Society (MDS) first published treatment recommendations for PD in 2002, which were updated in 2011 and 2016 [3]. The evidence base for the treatment of a range of NMS has increased substantially in recent years. Based on an extensive review of the literature, an update on the treatment of NMS was published in 2019 [3]. In many countries, there are also national PD treatment guidelines.

Against a background of the national and international PD treatment guidelines, the aim of this study was to investigate to which degree the recommendations are followed in the treatment of NMS in late-stage PD.

## 2. Methods

### 2.1. Participants and Recruitment

Participants were recruited in the southern region of Sweden through neurology departments and the municipality-based health care system. This cohort constitutes the Swedish part of the European multicenter study Care of Late-Stage Parkinsonism (CLaSP) [6, 21]. Late-stage PD was defined according to the inclusion criteria: HY stages IV-V (score range I–V, higher = worse) [4] in the medication “on” state and/or having a substantial need of help with activities of daily living (ADL), ≤50% on the Schwab and England ADL scale (score range 0–100, higher = better) [22]. Furthermore, participants had to have been diagnosed with PD since at least 7 years. Exclusion criteria were cognitive symptoms that started before the PD diagnosis as well as symptomatic parkinsonism, such as drug-induced parkinsonism or normal pressure hydrocephalus.

### 2.2. Procedure and Clinical Evaluation

An extensive data collection was carried out through home visits. A complete medication list was acquired from each participant. Nonmotor symptomatology was assessed with the NMSS (score range 0–360, higher = worse) [20]. Motor function was assessed with the Unified PD Rating Scale, UPDRS part III (score range 0–108, higher = worse) [23]. Cognition was assessed with the Mini-Mental State Examination (MMSE, score range 0–30, higher = better) [24]. Depressive symptoms were assessed with the Geriatric Depression Scale (GDS-30; score range 0–30, higher = worse) [25].

In the national as well as international PD treatment guidelines, recommendations for the pharmacological treatment of NMS have been given priority rankings. Priority according to the Swedish National Guidelines (i.e., the Swedish National Board of Health and Welfare's guidelines and the Swedish Movement Disorder Society's guidelines on diagnosis and treatment of PD) are categorized in the following way: priority 1–4 = recommended/should be used; priority 5–7 = can be used; priority 8–10 = can be used as an exception; research and education = should be used only in the frame of clinical studies; and non-do = should not be used [1, 2]. International treatment recommendations by the MDS are rated as clinically useful; possibly useful; investigational; and not useful [3].

Frequency and severity of the various NMS have in the present study been categorized in the following way: symptom present = individuals with a score of ≥1 on an item of the NMSS, scored 0–12 (higher = worse) and symptomatic individuals = individuals with a score of ≥6 on an item of the NMSS. For depressive symptoms, the GDS-30 cutoff in screening for depression (≥10 points) was used, where symptom present = 1–9 points and symptomatic individuals = ≥ 10 points. For cognitive symptoms, MMSE cutoffs were used: symptom present = below the cutoff for cognitive impairment of ≤23 points and symptomatic individuals = below the cutoff for possible dementia of ≤18 points on the MMSE.

Permission to use the questionnaires/assessment instruments used in this study has been obtained.

### 2.3. Ethical Considerations

The study was approved by the Swedish Ethical Review Authority, the Regional Ethical Review Board in Lund, Sweden (JPND HC-559-002). Written informed consent was obtained by the participants.

### 2.4. Statistical Analyses

Descriptive and clinical data are presented by median and first and third quartiles (q1–q3) and frequencies and percentages, as appropriate. All analyses were performed using IBM SPSS version 26.0 (IBM Corporation, Armonk, NY, USA).

## 3. Results

### 3.1. Demographic and Clinical Data

The study consisted of 107 patients in late-stage PD. Their median age was 78 years and their median disease duration was 15 years. The majority of the participants (79; 74%) were in HY stage IV, while 28 (26%) were in stage V. Independence in ADL was median 40% according to the Schwab & England Scale (Table 1). The NMS burden was median 91 points on the NMSS. All individuals exhibited the presence of NMS to various degrees and severities (Table 2).

Among symptomatic individuals (defined as ≥6 on item of the NMSS): for depressive symptoms, 37 of 63 (59%), who were above the ≥10 cutoff of the GDS for depression, were treated with antidepressants, 5 of 63 (8%) with serotonin-norepinephrine reuptake inhibitors (SNRIs)^Prio3^ (venlafaxine), 0 with tricyclic antidepressants (TCAs), 16 of 63 (25%) with noradrenergic and specific serotonergic antidepressants (NaSSAs)^No prio^ (mirtazapine), and 16 of 63 (25%) with selective serotonin reuptake inhibitors (SSRIs)^Prio8^. For hallucinations, 9 of 18 (50%) symptomatic individuals were treated with antipsychotics, 2 of 18 (11%) with clozapine^Prio3^, and 7 of 18 (39%) with quetiapine^Prio7^; for delusions, 5 of 13 (38%) were treated with antipsychotics, 3 of 13 (23%) with clozapine^Prio3^, and 2 of 13 (15%) with quetiapine^Prio7^. For dementia, among those who scored ≤18 on the MMSE, 9 of 27 (33%) were treated with acetylcholinesterase inhibitor rivastigmine^Prio4^, 1 of 27 (4%) was treated with acetylcholinesterase inhibitor donepezil^Prio4^, and 10 of 27 (37%) were treated with memantine^Prio9^. For OH, 11 of 19 (58%) with lightheadedness and 7 of 8 (88%) with fainting were treated with antihypotensives. For sialorrhea, 2 of 42 (5%) were treated with botulinum toxin^Prio4^; for constipation, 19 of 35 (54%) were treated with laxatives. For insomnia, 4 of 16 (25%) were treated with hypnotics; for daytime sleepiness, 1 of 29 (3%) was treated with psychostimulants (Table 3).

A more detailed description of pharmacological interventions recommended by the MDS in relation to their prescription in the current sample is given in Table 4. Among symptomatic individuals, for depression or depressive symptoms, 20 of 63 (32%) were treated with the dopamine-agonist pramipexole, rated as clinically useful according to MDS. No individuals were treated with TCA nortriptyline or desipramine; one was treated with amitriptyline (all alternatives were rated as possibly useful). As for SSRI/SNRI, 11 of 63 (17%) symptomatic individuals were treated with citalopram, 4 of 63 (6%) were treated with sertraline, none were treated with paroxetine, 1 of 63 (2%) was treated with fluoxetine (all alternatives rated as possibly useful), and 5 of 63 (8%) were treated with venlafaxine (rated as clinically useful). For lightheadedness, 3 of 19 (16%) symptomatic individuals were treated with fludrocortisone, 7 of 19 (37%) with midodrine, and none were treated with droxidopa (all alternatives rated as possibly useful). For fainting, no symptomatic individuals were treated with fludrocortisone or droxidopa, while 5 of 8 (63%) were treated with midodrine. For insomnia, 1 of 16 (6%) symptomatic individuals was treated with rotigotine (possibly useful), 3 (19%) with eszopiclone (possibly useful), and 1 (6%) with melatonin (possibly useful). For pain, among symptomatic individuals, 2 of 33 (6%) were treated with oxycodone-naloxone prolonged release (possibly useful), and for fatigue, 4 of 40 (10%) were treated with rasagiline (possibly useful).

## 4. Discussion

Treating clinicians have the support of national as well as international PD guidelines in the treatment of nonmotor symptomatology. The present results confirm that NMS are common and pronounced in late-stage PD and these analyses suggest that there may be a need for clinicians to further screen for and treat NMS in late-stage PD.

In the CLaSP project, which investigated the effect of specialist recommendations on therapy, it was concluded that clinicians who care for late-stage PD patients should be aware of the frequent occurrence of neuropsychiatric symptoms in this group [19]. This is in line with previous research, showing a high prevalence of neuropsychiatric symptoms in the advanced stages of the disease [17], including an increased risk of depression and psychosis with disease duration and of anxiety with increased cognitive impairment [26]. If depressive symptoms occur only during “off” periods, adjustment of antiparkinsonian medication is required [17]. Therefore, to determine when depressive symptoms occur and to adjust and optimize dopaminergic treatment is an important first step in the treatment of depression in PD.

The present analyses showed that for depressive symptoms, 41% of symptomatic individuals (i.e., individuals scoring above the established cutoff of ≥10 on the GDS-30 in screening for depression) were not prescribed any antidepressants. Treatment options for depression that were given the highest priority, i.e., rated as clinically useful in the international PD treatment guidelines, were used only in some cases among the symptomatic individuals of this sample. Dopamine agonist pramipexole, rated as clinically useful in the treatment of depressive symptoms in PD, was prescribed in 17 of 37 (46%) cases where the symptom was present but only in 20 of 63 (32%) cases among the more severely symptomatic individuals (Table 4). However, the indication for prescribing dopamine-agonist pramipexole may not have primarily been the treatment of depressive symptoms. Furthermore, venlafaxine, which has also been rated as clinically useful in the treatment of depressive symptoms, was used only in 1 of 37 (3%) cases where the symptom was present and in 5 of 63 (8%) cases in the more severely symptomatic group. Meanwhile, SSRIs^Prio8^, with very low priority in the national guidelines, was prescribed to 21 of the 51 (41%) individuals of the whole cohort who were on antidepressants, while only 6 (12%) were prescribed SNRIs^Prio3^ (e.g., venlafaxine), which have a high priority ranking. NaSSAs (e.g., mirtazapine), which have not been given a priority ranking, were the most commonly prescribed class of antidepressants, prescribed to 23 of 51 (45%) individuals (Table 3). However, part of the indication for prescribing mirtazapine may have been concurrent sleep disturbances.

In the total cohort of 107 individuals, 29 (27%) were prescribed antipsychotics. The efficacy of clozapine in the treatment of psychotic symptoms in PD has been confirmed in several studies [27]. Clozapine^Prio3^ was prescribed to 9 of 29 (31%) individuals, while quetiapine^Prio7^ was prescribed to 20 (69%) (Table 3). As clozapine in the international guidelines has been given the highest rating, clinically useful, while quetiapine was given the rating possibly useful, there may be reason to reconsider which of these medications to prescribe. There may to date be less evidence for the efficacy of quetiapine than clozapine, though a clear advantage with quetiapine over clozapine is easier handling without the need for strict blood monitoring [27]. Only half (9 of 18) of the individuals assessed with more pronounced hallucinations (≥6 on NMSS item, scored 0–12) and 5 of 13 (38%) with more pronounced delusions were prescribed antipsychotics (Table 3). This indicates that there may be a large number of individuals with a substantial symptom burden within this area who are not prescribed any treatment. This may be due to trouble with side effects of the medication such as confusion, sedation, OH, and the risk of agranulocytosis [27], though the present results suggest that it may be important to be aware of these symptoms and attempt to treat them, at least when they become bothersome for the patient. There may be different reasons for the lack of this; one may be not adequate screening of these symptoms in a clinical setting, and another may be underreporting of these symptoms by patients and informal caregivers due to possible embarrassment for such symptoms. Furthermore, concomitant cognitive impairment may contribute to patient loss of insight concerning these symptoms [28–30].

In many cases, it may be sufficient to reduce the dopaminergic therapy and switch to levodopa monotherapy in order to suppress hallucinations and delusions sufficiently. Antidementives are sometimes used as an alternative to antipsychotics when hallucinations are relatively mild. The goal does not have to be to eliminate the hallucinations completely; it is primarily to eliminate hallucinations that disturb the patient.

When it comes to cognitive impairment, another very common NMS in late-stage PD, 60 (58%) of the total sample scored below the general cutoff for cognitive impairment of ≤23 points on the MMSE, and about a quarter of the sample, 27 (26%), scored ≤18 on the MMSE, which may indicate a more substantial cognitive impairment or dementia, i.e., here referred to as symptomatic individuals (Table 1). Acetylcholinesterase inhibitors have been given priority four in the national guidelines, and rivastigmine has been given the rating clinically useful in the international PD treatment guidelines, while donepezil, also priority four in the national guidelines, has been given the rating possibly useful in the international guidelines. In the present cohort, the most common medication for cognitive impairment and dementia was memantine^Prio9^, prescribed to 22 of 60 (37%) individuals with symptom present and to 10 of 27 (37%) individuals with more severe symptomatology. This probably reflects the treatment tradition for this group of patients in Sweden presently. Rivastigmine^Prio4^ was prescribed to 15 (14%) in the total sample and to only 9 of 27 (33%) of the symptomatic individuals (Table 3). This clearly indicates an undertreatment of these common symptoms in this rather large sample of patients in late-stage PD. As cognitive impairment/dementia are among PD symptoms that affect not only the patients but, to a high degree, also their informal caregivers' QoL and perceived caregiver burden [31], it is important to recognize these symptoms and to evaluate if symptomatology can be improved with recommended pharmacological therapy. The fact that these therapies often have a relatively weak effect may contribute to some physicians not prescribing them.

OH is yet another common and highly debilitating and troublesome NMS in late-stage PD, which is also a risk factor for falls and fractures in PD [32]. In a previous levodopa test study on the current sample, 21 of 30 (70%) patients exhibited OH, defined as a decrease in blood pressure of ≥20 mm Hg systolic or ≥10 mm Hg diastolic, after three minutes of standing [2, 33]. In this cohort of 107 patients in late-stage PD, 27 (25%) were prescribed antihypotensives. Midodrine^Prio3^ was prescribed to 7 of 19 (37%) individuals with a more severe symptomatology of lightheadedness and to 5 of 8 (63%) among those with more severe problems with fainting (Table 3). As motor problems with gait, freezing of gait, and postural instability are very common in late-stage PD [7, 34, 35], the additional risk of falls related to OH should be reduced by adequate pharmacological treatment. Also, fatigue, cognitive difficulties, dizziness, and double vision are common side effects with low blood pressure. However, the main difficulty is to balance the treatment so that the patient does not, at other times, get a too high blood pressure.

Sleep disturbances are yet another category of NMS common in late-stage PD, affecting a large proportion (30–66%) of the sample to some degree (Table 2). Of the total sample, 28 (26%) were prescribed hypnotics. Only one quarter (4 of 16; 25%) of those with more severe symptomatology (≥6 on item 5 of the NMSS, insomnia) were prescribed any hypnotic/sedative/insomnia medication. However, there are several different reasons for sleep disturbances in PD, e.g., problems with “off” time rigidity/bradykinesia, nocturia, and nightmares/hallucinations, which may need other specific treatments.

Patients in late-stage PD are often prescribed several medications and have severe PD symptomatology with a large motor and nonmotor burden. Even though potential side effects on PD features such as OH sometimes may limit treatment options [6], therapeutic gains may be reached also in the vulnerable group of patients in late-stage PD [36]. Thoroughly screening the patients' symptomatology and creating individualized approaches to therapy, taking possible side effects into consideration, has been recommended [37]. This approach is likely important throughout all stages of PD, not least in the late stage.

There may be a proportion of the sample whose NMS symptomatology responded so well to pharmacological therapy that they did not receive higher scores on the NMSS. Furthermore, with mild symptomatology, it is possible that no pharmacological treatment was given. There may also be individual factors, such as cognition, that influence the way patients respond to questions in the assessment of NMS. However, being a clinician-administered interview-based rating scale, the NMSS scoring is rated in dialogue with the patient and, in some cases, with his/her informal caregiver, assessing both the frequency and severity of each symptom.

An interesting group may be those who do not have any symptoms, though receive treatment. When it comes to depressive symptoms, 51 individuals (48%) of the total sample were prescribed antidepressants, of which 37 scored above the established GDS-cutoff in screening for depression, while the remaining individuals who received antidepressants scored somewhere below the cutoff for depression, which may indicate that there is a treatment effect.

However, in the present study, the individuals with more severe symptomatology who do not have any medication indicate an undertreatment throughout a large range of NMS. There may be different reasons for this, such as medication side effects, interactions with other drugs, comorbidities, and the frailty of the late-stage patient [37]. Another reason could be that the patient due to the severity of the late stage of the disease has difficulty getting to the specialist clinic [6, 10] and is not treated by a physician with a special interest in and knowledge of movement disorders. Many patients have several different NMS, and a specific treatment for each of them could lead to a large number of pharmacological therapies, with the accompanying risk of interactions and side effects. This might limit the possibility of intervening with NMS in some patients. However, since many of the commonly prevalent NMS in late-stage PD are potentially treatable, more attention should be given to the assessment and treatment of NMS in this severely affected population [15].

There is a large variety in treatment strategies for late-stage PD across different countries. Consequently, there is a need to develop guidelines for management in late-stage PD to ascertain that these patients receive the best possible care [6]. This should likely be done both nationally and internationally.

### 4.1. Strengths, Limitations, and Future Perspectives

Through home visits, we successfully included a large cohort of 107 patients in the late and most severe stage PD, a group often excluded in research, whose situation probably could be improved. In order to assess the prevalence and severity of the various NMS, cutoffs were made to distinguish between individuals with symptoms present, i.e., all individuals who exhibited a symptom and individuals with more severe symptomatology. As there are no recommended cutoffs of the NMSS, we attempted to make a cutoff at ≥ 6 points on each item, on a scale from 0 to 12, in order to distinguish individuals with presumed moderate to severe symptomatology. As for the GDS-30 and the MMSE, generally established cutoffs were used. A hope for the future is improved possibilities to detect NMS, as well as more effective treatment methods with less tendency to side effects and interactions.

Reasons for why overall there is relatively limited use of recommended therapies may include lack of treatment effect, side effects, risk of interactions with other drugs, and patient and informal caregiver refusal of treatment. In addition, there is reason to believe that there is an undertreatment which may be due to the unawareness of treatment options and recommendations.

Future studies on adherence to NMS treatment guidelines in other national contexts would give valuable information on possible differences depending on location and cultural differences.

## 5. Conclusions

Against a background of the national and international treatment guidelines, individual treatment may be reviewed and optimized throughout all stages of PD, thus improving symptomatology and the overall situation for patients and their families. The present results confirm that NMS are common and pronounced in late-stage PD and the analyses suggest a need for clinicians to further screen for and treat NMS in late-stage PD. Optimizing treatment of NMS according to the national and international treatment guidelines may improve symptomatology and enhance QoL in late-stage PD.

## Figures and Tables

**Table 1:** Demographics and clinical symptomatology in late-stage PD (*n* = 107).

	Total cohort	Missing
Gender, *n* (%)		—
Male	62 (58%)	
Female	45 (42%)	
Age, median (q1–q3)	78 (73–84)	—
Age at onset (years), median (q1–q3)	63 (55–71)	—
PD duration (years), median (q1–q3)	15 (11–19)	—
LEDD, median (q1–q3)	798 (560–998)	—
Partner (yes), *n* (%)	65 (61%)	—
Disease severity (Hoehn and Yahr), *n* (%)		—
Stage IV	79 (74%)	
Stage V	28 (26%)	
Independence ADL (%) (Schwab and England), median (q1–q3)	40 (30–50)	—
Motor function (UPDRS III), median (q1–q3)	40 (29–53)	—
Nonmotor burden (NMSS, total score), median (q1–q3)	91 (55–128)	2
Cognitive impairment		
MMSE total score, median (q1–q3)	22 (18–27)	4
Proportion ≤23, *n* (%)	60 (58%)	
Proportion ≤18, *n* (%)	27 (26%)	
Depressive symptoms		
GDS-30, median (q1–q3)	11 (8–16)	7
Proportion ≥10, *n* (%)	63 (63%)	

PD, Parkinson's disease; q1–q3, first and third quartiles; LEDD, levodopa equivalent daily dose; HY, Hoehn and Yahr staging scale (score range I–V, higher = worse); S&E, Schwab and England ADL scale (score range 0–100, higher = better); UPDRS III, Unified PD Rating Scale, part III = motor examination (score range 0–108, higher = worse); MMSE, Mini-Mental State Examination (score range 0–30, higher = better); GDS-30, Geriatric Depression Scale (score range 0–30, higher = worse).

**Table 2:** Prevalence and severity of nonmotor symptoms in late-stage PD (*n* = 105).

	NMSS score (median, q1–q3)	≥1 symptom present (*n*, %)	≥6 symptomatic individuals (*n*, %)
D1: cardiovascular	1 (0–4)		
(1) Lightheadedness	1 (0–4)	54 (51%)	19 (18%)
(2) Fainting	0 (0-0)	21 (20%)	8 (8 %)
D2: sleep/fatigue	9 (4–17)		
(3) Daytime sleepiness	2 (0–6)	63 (60%)	29 (28%)
(4) Fatigue	4 (0–8)	69 (66%)	40 (38%)
(5) Difficulty falling asleep	0 (0–3)	31 (30%)	16 (15%)
(6) Restless legs	0 (0–4)	38 (36%)	20 (19%)
D3: mood/apathy	13 (4–27)		
(7) Lost interest in surroundings	0 (0–4)	47 (45%)	25 (24%)
(8) Lack motivation	3 (0–6)	63 (60%)	36 (34%)
(9) Feel nervous	0 (0–2)	34 (32%)	17 (16%)
(10) Seem sad	0 (0–4)	53 (50%)	26 (25%)
(11) Flat moods	4 (0–6)	67 (64%)	27 (26%)
(12) Difficulty experiencing pleasure	0 (0–2)	34 (32%)	18 (17%)
D4: perceptual problems/hallucinations	4 (0–10)		
(13) Hallucinations	0 (0–4)	49 (47%)	18 (17%)
(14) Delusions	0 (0-1)	27 (26%)	13 (12%)
(15) Double vision	0 (0–3)	36 (34%)	18 (17%)
D5: attention/memory	9 (2–23)		
(16) Concentration	2 (0–6)	61 (58%)	36 (34%)
(17) Forget things or events	2 (0–6)	66 (63%)	33 (31%)
(18) Forget to do things	4 (0–12)	69 (66%)	43 (41%)
D6: gastrointestinal	9 (4–16)		
(19) Saliva	4 (0–8)	65 (62%)	42 (40%)
(20) Swallowing	0 (0–4)	44 (42%)	25 (24%)
(21) Constipation	2 (0–6)	58 (55%)	35 (33%)
D7: urinary	12 (4–24)		
(22) Urgency	4 (0–12)	74 (70%)	50 (47%)
(23) Frequency	0 (0–8)	53 (50%)	35 (33%)
(24) Nocturia	4 (0–12)	71 (68%)	46 (44%)
D8: sexual dysfunction	12 (0–12)		
(25) Interest in sex	0 (0–4)	29 (28%)	18 (17%)
(26) Problems having sex	8 (0–12)	60 (57%)	57 (54%)
D9: miscellaneous	12 (4–20)		
(27) Pain	1 (0–8)	54 (51%)	33 (31%)
(28) Taste or smell	4 (0–12)	63 (60%)	49 (47%)
(29) Weight change	0 (0–2)	34 (32%)	13 (12%)
(30) Excessive sweating	0 (0–2)	27 (26%)	10 (10%)
NMSS, total score (median, q1–q3)	91 (55–128)		

Values are presented as median and first and third quartiles (q1–q3). NMSS, Nonmotor Symptoms Scale (0–360, higher = worse); D, domain (severity × frequency of each item of the domain are added together: 0–12, higher = worse). Each item is scored: severity (0–3, higher = worse) and frequency (1–4, higher = worse).

**Table 3:** Pharmacological treatment of nonmotor symptoms in late-stage PD in relation to the recommendations of the priority rating in the Swedish National Board of Health and Welfare's guidelines and in the Swedish Movement Disorder Society's guidelines (*n* = 107).

	Total cohort	Symptom present^a^(n, %)	Symptomatic individuals^b^(n, %)
Depressive symptoms^c^			
No antidepressant medication, n (%)	56 (52%)	23 of 37 (62%)	26 of 63 (41%)
Antidepressant medication, *n* (%)	51 (48%)	14 of 37 (38%)	37 of 63 (59%)
SNRI^*∗*Prio3^ (venlafaxine)	6 of 51 (12%)	1 of 37 (3%)	5 of 63 (8%)
TCA^*∗*Prio4^	1 of 51 (2%)	1 of 37 (3%)	0 of 63 (0%)
NaSSA^No prio^ (mirtazapine)	23 of 51 (45%)	7 of 37 (19%)	16 of 63 (25%)
SSRI^*∗∗*Prio8^	21 of 51 (41%)	5 of 37 (14%)	16 of 63 (25%)
Anxiety^d^			
Anxiolytics	15 (14%)	5 of 34 (15%)	1 of 17 (6%)
Psychotic symptoms^e^			
Antipsychotics	29 (27%)		
Clozapine^*∗*Prio3^	9 of 29 (31%)		
Quetiapine^*∗∗*Prio7^	20 of 29 (69%)		
Hallucinations			
Antipsychotics		16 of 49 (33%)	9 of 18 (50%)
Clozapine^*∗*Prio3^		6 of 49 (12%)	2 of 18 (11%)
Quetiapine^*∗∗*Prio7^		10 of 49 (20%)	7 of 18 (39%)
Delusions			
Antipsychotics		12 of 27 (44%)	5 of 13 (38%)
Clozapine^*∗*Prio3^		6 of 27 (22%)	3 of 13 (23%)
Quetiapine^*∗∗*Prio7^		6 of 27 (22%)	2 of 13 (15%)
Cognitive impairment			
Dementia^f^ (MMSE ≤ 23, n = 60/≤18, n = 27)			
Acetylcholinesterase inhibitors^*∗*Prio4^			
Rivastigmine^*∗*Prio4^, n (%)	15 (14%)	14 of 60 (23%)	9 of 27 (33%)
Donepezil^*∗*Prio4^, n (%)	4 (4%)	3 of 60 (5%)	1 of 27 (4%)
Memantine^*∗∗∗*Prio9^, n (%)	23 (22%)	22 of 60 (37%)	10 of 27 (37%)
Autonomic dysfunction			
Orthostatic hypotension^g^			
Antihypotensives	27 (25%)		
Midodrine^*∗*Prio3^	16 (15%)		
Fludrocortison^*∗∗*Prio5^	6 (6%)		
Others	12 (11%)		
Droxidopa^*∗∗∗*Prio8^	1 (1%)		
Etilefrin^*∗*Prio3^	7 (7%)		
Lightheadedness			
Antihypotensives		20 of 54 (37%)	11 of 19 (58%)
Midodrine^*∗*Prio3^		10 of 54 (19%)	7 of 19 (37%)
Fludrocortison^*∗∗*Prio5^		4 of 54 (7%)	3 of 19 (16%)
Others		9 of 54 (17%)	4 of 19 (21%)
Droxidopa^*∗∗∗*Prio8^		0	0
Etilefrin^*∗*Prio3^		6 of 54 (11%)	2 of 19 (11%)
Fainting			
Antihypotensives		12 of 21 (57%)	7 of 8 (88%)
Midodrine^*∗*Prio3^		7 of 21 (33%)	5 of 8 (63%)
Fludrocortison^*∗∗*Prio5^		3 of 21 (14%)	0
Others		4 of 21 (19%)	2 of 8 (25%)
Droxidopa^*∗∗∗*Prio8^		0	0
Etilefrin^*∗*Prio3^		3 of 21 (14%)	2 of 8 (25%)
Sialorrhea^h^			
Botulinum toxin^*∗*Prio4^, n (%)	4 (4%)	2 of 65 (3%)	2 of 42 (5%)
Constipation^i^			
Laxatives, n (%)	54 (50%)	33 of 58 (57%)	19 of 35 (54%)
Urinary urgency/frequency/nocturia^j^			
Peripheral anticholinergics^*∗∗*Prio6^			
Detrusitol	0	0	0
Emselex	0	0	0
Vesicare	1 (1%)	1 of 91 (1%)	1 of 71 (1%)
Betmiga	3 (3%)	2 of 91 (2%)	2 of 71 (3%)
Botulinum toxin injection bladder^*∗∗*Prio7^	NAv	NAv	NAv
Sleep disturbances/disorders			
Insomnia^k^			
Hypnotics/sedative/insomnia drugs, n (%)	28 (26%)	7 of 31 (23%)	4 of 16 (25%)
Daytime sleepiness^l^			
Psychostimulant drugs, n (%)	1 (1%)	1 of 63 (2%)	1 of 29 (3%)
Behavioral disorders			
Dopaminergic dysregulation syndrome			
Impulse control disorder			
Naltrexone^*∗∗∗*Prio9^	NAv	NAv	NAv

PD, Parkinson's disease; q1–q3, first and third quartiles; NMSS, Nonmotor Symptoms Scale (0–360, higher = worse); D, domain (severity × frequency of each item of the domain are added together; each item is scored 0–12 (higher = worse), 2 missing. MMSE, Mini-Mental State Examination (0–30, higher = better), 4 missing; GDS-30, geriatric depression scale (0–30, higher = worse), 7 missing; SSRI, selective serotonin reuptake inhibitor; NaSSA, noradrenergic and specific serotonergic antidepressants; SNRI, serotonin-norepinephrine reuptake inhibitors; TCA, tricyclic antidepressants. NAv, information nonavailable. ^a^Symptom present, individuals with score ≥1 on item of the NMSS scored 0–12 (higher = worse), if nothing else is stated. ^b^Symptomatic individuals include those who score above the cutoff of ≥6 (moderate–severe) on item of the NMSS, if nothing else is stated. ^c^Depressive symptoms based on GDS-30 cutoff for depression (≥10 points): symptom present 1–9 points, symptomatic individuals ≥10 points. ^d^Anxiety score refer to (NMSS item 9) feel nervous, as no other specific data for anxiety was collected. ^e^Psychotic symptoms scores refer to item 13 (hallucinations) and item 14 (delusions) of the NMSS. ^f^Dementia as assessed by the MMSE, cutoffs cognitive impairment ≤23/dementia ≤18 MMSE. ^g^Orthostatic hypotension based on item 1 (lightheadedness) and item 2 (fainting) of the NMSS. ^h^Siallorhea based on item 18 of the NMSS. ^i^Constipation based on item 21 of the NMSS. ^j^Urinary urgency/nocturia based on domain 7 of the NMSS. ^k^Insomnia based on item 5 of the NMSS. ^l^Daytime sleepiness based on item 3 of the NMSS. Priority according to the Swedish national guidelines (the Swedish National Board of Health and Welfare's guidelines and the Swedish movement disorder society's guidelines on diagnosis and treatment of PD): ^*∗*^Priority 1–4 (recommended/should be used), ^*∗∗*^Priority 5–7 (can be used), ^*∗∗∗*^Priority 8–10 (can be used as an exception), research and education (Swedish FoU): should be used only in the frame of clinical studies, non-do: should not be used.

**Table 4:** Pharmacological treatment of nonmotor symptoms in late-stage PD in relation to the recommendations of the International Parkinson and Movement Disorder Society (*n* = 107).

	Total cohort	Symptom present^a^(*n*, %)	Symptomatic individuals^b^(*n*, %)
Neuropsychiatric symptoms			
Depression and depressive symptoms^c^			
Dopamine agonists			
Pramipexole^d Clin useful^	39 (36%)	17 of 37 (46%)	20 of 63 (32%)
Tricyclic antidepressants			
Nortriptyline^Pos useful^	0	0	0
Desipramine^Pos useful^	0	0	0
Amitriptyline^Pos useful^	1 (1%)	0	1 of 63 (2%)
SSRI/SNRI			
Citalopram^Pos useful^	14 (13%)	3 of 37 (8%)	11 of 63 (17%)
Sertraline^Pos useful^	6 (6%)	2 of 37 (5%)	4 of 63 (6%)
Paroxetine^Pos useful^	0	0	0
Fluoxetine^Pos useful^	1 (1%)	0	1 of 63 (2%)
Venlafaxine^Clin useful^	6 (6%)	1 of 37 (3%)	5 of 63 (8%)
Anxiety and anxiety symptoms^e^			
Apathy			
Dopamine agonists			
Piribedil^Pos useful^	0	0	0
Acetylcholinesterase inhibitors			
Rivastigmine^f Pos useful^	15 (14%)	14 of 60 (23%)	9 of 27 (33%)
Psychosis^g^			
Clozapine^Clin ueful^	9 (8%)		
Quetiapine^Pos useful^	20 (19%)		
Pimavanserin^*∗*Clin useful^	0	0	0
Hallucinations			
Clozapine^Clin ueful^		6 of 49 (12%)	2 of 18 (11%)
Quetiapine^Pos useful^		10 of 49 (20%)	7 of 18 (39%)
Delusions			
Clozapine^Clin ueful^		6 of 27 (22%)	3 of 13 (23%)
Quetiapine^Pos useful^		6 of 27 (22%)	2 of 13 (15%)
Dementia^h^			
Acetylcholinesterase inhibitors			
Donepezil^Pos useful^	4 (4%)	3 of 60 (5%)	1 of 27 (4%)
Rivastigmine^Clin useful^	15 (14%)	14 of 60 (23%)	9 of 27 (33%)
Galantamine^Pos useful^	0	0	0
Autonomic dysfunction			
Drooling^i^			
Glycopyrrolate^Pos useful^	0	0	0
Botulinum toxin^Clin useful^	4 (4%)	2 of 65 (3%)	2 of 42 (5%)
Orthostatic hypotension^j^			
Fludrocortisone^Pos useful^	6 (6%)		
Midodrine^Pos useful^	16 (15%)		
Droxidopa^Pos useful^	1 (1%)		
Light headedness			
Fludrocortisone^Pos useful^		4 of 54 (7%)	3 of 19 (16%)
Midodrine^Pos useful^		10 of 54 (19%)	7 of 19 (37%)
Droxidopa^Pos useful^		0	0
Fainting			
Fludrocortisone^Pos useful^		3 of 21 (14%)	0
Midodrine^Pos useful^		7 of 21 (33%)	5 of 8 (63%)
Droxidopa^Pos useful^		0	0
Urinary dysfunction^k^			
Frequency, urgency and/or urge incontinence			
Solifenacin^Pos useful^	3 (3%)	3 of 58 (5%)	0
Erectile dysfunction			
Sexual dysfunction			
Sildenafil^Clin useful^	0	0	0
Gastrointestinal dysfunction			
Constipation^l^			
Macrogol^Pos useful^	54 (50%)	33 of 58 (57%)	19 of 35 (54%)
Lubiprostone^*∗*Pos useful^	0	0	0
Anorexia, nausea and vomiting associated with L-dopa or DA treatment			
Domperidone^m Pos useful^	2 (2%)	NAv	NAv
Disorders of sleep and wakefulness			
Sleep fragmentation and insomnia^n^			
Insomnia			
Dopamine agonists			
Rotigotine^o Pos useful^	2 (2%)	2 of 31 (6%)	1 of 16 (6%)
Hypnotics			
Eszopiclone^Pos useful^	15 (14%)	5 of 31 (16%)	3 of 16 (19%)
Melatonin			
3–5 mg^Pos useful^	2 (2%)^p^	1 of 31 (3%)	1 of 16 (6%)
Rapid eye movement sleep behavior disorder			
Excessive daytime sleepiness^q^			
Psychoactive drugs			
Modafinil^Pos useful^	1 (1%)	1 of 63 (2%)	0
Others			
Pain^r^			
Oxycodone-naloxone prolonged release^Pos useful^	4 (4%)	2 of 54 (4%)	2 of 33 (6%)
Fatigue^s^			
MAO-B inhibitors			
Rasagiline^Pos useful^	14 (13%)	10 of 69 (14%)	4 of 40 (10%)

PD, Parkinson's disease; q1–q3, first and third quartiles; MMSE, mini-mental state examination (0–30, higher = better); GDS-30, geriatric depression scale (0–30, higher = worse); SSRI, selective serotonin reuptake inhibitor; NaSSA, noradrenergic and specific serotonergic antidepressants; SNRI, serotonin-norepinephrine reuptake inhibitors; TCA, tricyclic antidepressants; L-dopa, levodopa; DA, dopamine agonists; MAO-B, monoamine oxidase B. NAv, information nonavailable. ^*∗*^Not available in Sweden. Table includes treatment rated by the International Parkinson and Movement Disorder Society as clinically useful and possibly useful, while investigational and not useful treatments are not listed. ^a^Symptom present, individuals with score ≥1 on item of the NMSS scored 0–12 (higher = worse), if nothing else is stated. ^b^Symptomatic individuals include those who score above the cutoff of ≥6 (moderate–severe) on item of the NMSS, if nothing else is stated. ^c^Depressive symptoms based on GDS-30 cutoff for depression (≥10 points): symptom present 1–9 points, symptomatic individuals ≥10 points. ^d^Indication not known. ^e^Anxiety score refer to (NMSS item 9) feel nervous, as no other specific data for anxiety was collected. ^f^Indication not known. ^g^Psychotic symptoms scores refer to item 13 (hallucinations) and item 14 (delusions) of the NMSS. ^h^Dementia as assessed by the MMSE, cutoffs cognitive impairment ≤23/dementia ≤18 MMSE. Treatment alternatives for cognitive impairment are all investigational and hence not listed. ^i^Siallorhea based on item 18 of the NMSS. ^j^Orthostatic hypotension refer to item 1 (lightheadedness) and item 2 (fainting) of the NMSS. ^k^Urinary urgency/nocturia based on domain 7 of the NMSS. ^l^Constipation based on item 21 of the NMSS. ^m^Indication not known. ^n^Insomnia based on item 5 of the NMSS. ^o^Indication not known. ^p^Both individuals on Melatonin 2 mg ^q^Daytime sleepiness based on item 3 of the NMSS. ^r^Pain based on item 27 of the NMSS. ^s^Fatigue based on item 4 of the NMSS.

## Data Availability

The data used to support the findings of this study are available from the corresponding author upon request.
